# New products

**DOI:** 10.1155/S1463924699000085

**Published:** 1999


Journal of Automated Methods & Management in Chemistry, Vol. 21, No. 2 (March–April 1999) pp. 45–63

### New products

### Automated SPE extraction of tricyclic antidepressant drugs from serum

With the widespread clinical use of antidepressant drugs comes the need to accurately measure therapeutic levels to determine proper dosage, as well as check for toxic levels and patient compliance. More and more laboratories are shifting their therapeutic drug monitoring from liquid liquid extraction to solid phase extraction. A large clinical laboratory has automated the process of extracting these drugs from serum using the RapidTrace SPE Workstation.

Prior to the extraction, 1 ml of serum is diluted with 2 ml of phosphate buffer and 0.1 ml of internal standard. The sample is vortexed and centrifuged, then decanted into a 13 × 100 mm test tube to remove any particulates. Once all samples are prepared, they are placed in the RapidTrace with 3 ml syringe barrel HCX columns (International Sorbent Technology).

After the extraction is complete, the 3 ml sample eluate is evaporated to dryness and reconstituted with the mobile phase. Samples are analysed by HPLC.

Control samples were prepared with four common antidepressant drugs (Amitriptyline HCL, Nortriptyline HCL, Imipramine HCL, Desipramine HCL). For this study, samples containing these drugs were run for five consecutive days. As demonstrated in table 1, this extraction technique yielded excellent day to day reproducibility.

This method uses Zymark's RapidTrace® SPE Workstation to perform the sample extraction through the SPE column. Treatment of the sample before and after the extraction is described, the automated steps of the extraction are outlined, and recovery data are shown.

The RapidTrace SPE Workstation was designed to provide high sample throughput, reproducible results and the tools for SPE methods development. A RapidTrace Workstation contains up to 10 individual modules, which run a single procedure or multiple procedures across the workstation. The high sample throughput is obtained through 'batch processing' of samples, allowing 40–100 samples to be extracted per hour. A high level of accuracy and reproducibility is achieved by precisely controlling the flow rates for all steps of the SPE process: column conditioning, loading and sample, rinsing the column, and eluting the components of interest. The RapidTrace for Windows$^{TM}$ software can facilitate methods development allowing the user to write one procedure, then easily varying parameters, e.g. column type, reagents and flow rates. The RapidTrace Workstation is ideal in the Drug Metabolism/Pharmacokinetic, Toxicology or Forensic Testing Lab.

### Robotic handling for dangerous substances—an unbeatable combination

When it comes to weighting equipment, METTLER TOLEDO is unbeatable, says Peerless Systems, which has an enviable reputation for manufacturing robotic systems to handle radioactive and other dangerous substances injurious to human health.

Since its inception in 1982, the Washington, Tyne and Wear, based company has always employed METTLER TOLEDO's advanced weighting technology when supplying equipment for use in radioactive environments.

"One of our specialities is building systems for duties in environmentally unfriendly locations", explained managing director, Moira Lamb. "We have never used anything other than METTLER TOLEDO balances because of their consistent quality and reliability", she remarked, adding that some of its original systems still in regular use utilize balance components that are now 15 or 16 years old.

Over the years, Peerless has purchased a succession of PE, PM, PG, SG and other METTLER TOLEDO models which have been stripped down and split into two separate stainless steel enclosures: one housing the weigh cell, the other housing the major electronics.

*Table 1.*

| | *Amitriptyline (ng/ml)* | *Nortriptyline (ng/ml)* | *Imipramine (ng/ml)* | *Desipramine (ng/ml)* |
|---|---|---|---|---|
| Day 1 | 93.0 | 102.7 | 113.3 | 104.3 |
| Day 2 | 95.6 | 104.2 | 112.2 | 104.6 |
| Day 3 | 94.5 | 104.8 | 113.6 | 103.4 |
| Day 4 | 92.8 | 106.7 | 115.2 | 108.6 |
| Day 5 | 96.8 | 103.4 | 118.6 | 109.1 |
| | mean = 94.54 | mean = 104.36 | mean = 114.58 | mean = 106 |
| Target value | 95.9 | 102.7 | 112.3 | 106.3 |
| Average recovery (%) | 98.5 | 102.6 | 101.9 | 99.7 |
| C.V. (%) | 1.8 | 1.5 | 2.2 | 2.5 |

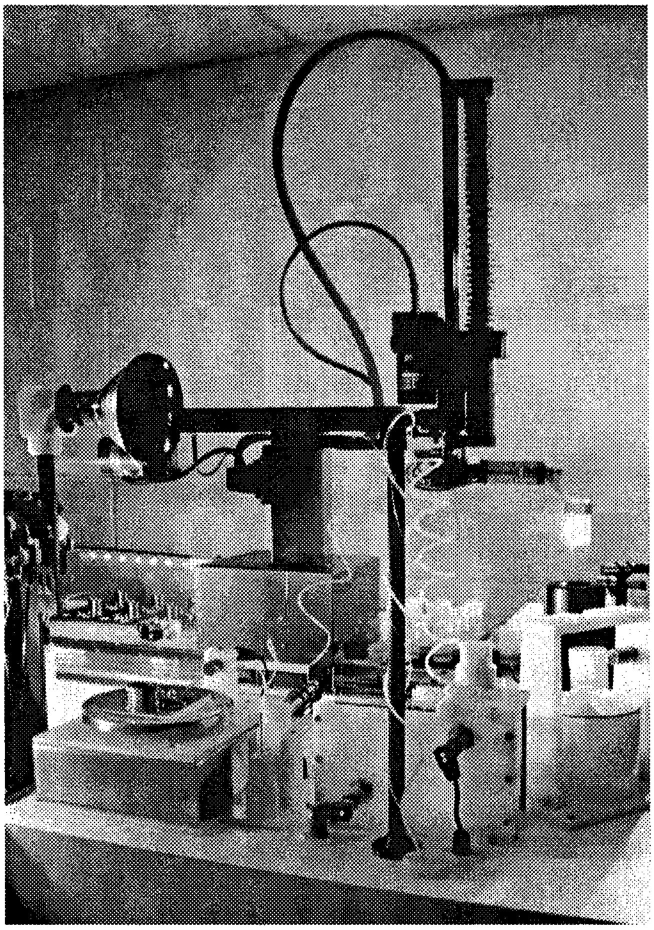

*A robotic handling system for radioactive substances incorporating METTLER TOLEDO's weigh cell and measuring components.*

The two units are interconnected by up to 12 m of screened cable. This configuration allows only the weigh cell unit, which has been designed to minimize the ingress of particular matter, to be exposed to hazardous environments, while the electronics can be located in a remote, clean area. Numerous systems have been supplied to the nuclear, pharmaceuticals and chemical industries.

"We have established a very specialized niche market and as far as we know, nobody else makes systems like ours", she said. "METTLER TOLEDO has played an important part in our development and will no doubt continue to be our preferred supplier of balances for many years to come."

*For further information, contact: Roger Norton, Norton Harris Partners, 26 Church Street, Wilmslow, Cheshire SK9 1AU, UK. Tel: 01625 529293, Fax: 01625 539737.*

**New chrom perfect for HP gas chromatograms data sheet now available from justice laboratory software (UK)**

The superior data integration and report generation capabilities of the Chrom Perfect Chromatography Data System have now been combined with instrument control features for use with Hewlett-Packard 6890 and 5890 gas chromatographs. Chrom Perfect eliminates the limitations placed on the lab by the Hewlett-Packard Interface Bus (HPIB). A single Chrom Perfect data system can acquire data from multiple instruments from any manufacturer, while digitally acquiring and controlling an HP 6890 and/or 5890 from the same PC platform.

The basic system can handle two chromatographs per PC. Upgrades allow you to handle four or eight GCs with a total of 16 detectors. Each GC can have its own method, printer and data directory. You see real-time chromatographs for all active detectors. A simulation of the GC front panel allows you to control the instrument from your PC. You can remotely control all temperatures, pressures and set-points from your PC. The two-chromatograph version can be run in lap-top computers. It uses standard COM ports found in most lap-tops, no special cards are required.

Justice Laboratory Software (UK) offers the most comprehensive line of high technology software products for your analytical laboratory. From NT-based client server chromatography systems, to single user basic data systems, Justice Laboratory Software (UK) meets the demanding needs of chromatography applications. Our products offer multi-vendor instrument interfacing and distributed data processing for total integration of your lab.

*For more information, contact: Justice Laboratory Software (UK), Tel: 01337 828494, Fax: 01337 828 404, Email: ContactUs@JusticeUK.com, Web Site: WWW.JusticeUK.com*

**New size exclusion/gel permeation chromatography software now available from Justice Laboratory Software**

Justice Laboratory Software (UK) announces a full-featured Size Exclusion Chromatography (SEC) software package that is fully integrated with Chrom Perfect$^{TM}$ for Windows. With three modes of operation, the software allows you to retrieve a chromatogram collected by Chrom Perfect and produce SEC plots and reports interactively. It also allows you to produce reports and plots interactively after each run or by batch reprocessing a series of runs. Specifications come from a SEC method file that the chemist prepares ahead of time.

The SEC/GPC software package supports a wide variety of calibrations including: narrow standard, broad standard and universal calibration. The software fits the calibration curve with a polynomial up to degree 5.

This software can transfer plots and reports to other software, e.g. EXCEL, Lotus 1-2-3 or most word processing programs. It leaves SEC results in ASCII file format for transfer to your word processor or desktop publishing software. SEC provides a wide variety of calibrations and reports, and produces accurate and reproducible measurements of molecular weight.

Justice Laboratory Software (UK) offers the most comprehensive line of high technology software products for your analytical laboratory. From NT-based client server chromatography systems, to single user basic data systems, Justice Laboratory Software (UK) meets the demanding needs of chromatography applications. Our products offer multi-vendor instrument interfacing and distributed data processing for total integration of your lab.

*For more information, contact: Justice Laboratory Software (UK), Tel: 01337 828494, Fax: 01337 828 404, Email: ContactUs@JusticeUK.com, Web Site: WWW.JusticeUK.com*

### New specific interface allows simple, reliable measurements of mercury in natural gas

The P S Analytical Sir Galahad system has been specifically designed for the determination of mercury in gaseous environments. One of the important areas where this instrument can provide an immediate advantage is in the Natural Gas Industry.

Several major disasters have arisen due to damage caused by mercury contamination. Measuring the presence and form of mercury is therefore important to the industry. The Sir Galahad system offers significant advantages, since the sample itself is a natural gas of varying compositions often at high pressures, i.e. greater than 2500 psig, providing a truly representative sample in what can be seen as a difficult procedure.

P S Analytical has recently introduced a specifically designed pressure let-down system which provides the ideal interface between the high pressure gas lines and the Sir Galahad system. The PSA 10.537 Pressure Let-Down System is a compact unit that requires no services other than a simple link to the sample line. It can operate at pressures of up to 3000 psig and provides a reproducible sample at atmospheric pressure over the standard Amasil$^{TM}$ traps of the Sir Galahad. Depending on the contaminant level in the natural gas, the sampling period can be adjusted to provide an optimal measurement.

The samples are collected and the trap transferred to the static head of the Sir Galahad prior to measurement in the normal manner. Using the above technology, the PSA analytical team can quickly survey gas supply lines either on-shore or off-shore. This carefully designed

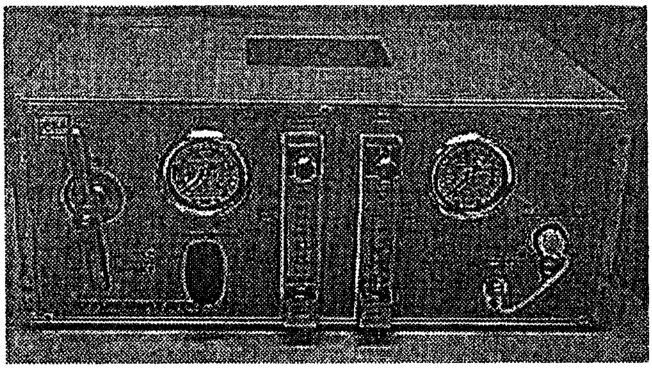

PSA Merlin | Excalibur PSA | Sir Galahad II

interface therefore provides the necessary and complete control of sampling prior to analysis.

Sir Galahad II is a registered trademark of P S Analytical.

For Further details on the Sir Galahad or the Pressure Let-Down System, please contact: Paul Stockwell, P S Analytical Ltd, Arthur House, Crayfields Industrial Estate, Main Road, Orpington, Kent BR5 3HP, UK.
Tel (Int): +44 (0)1689 891211;
Fax (Int): +44 (0)1689+ 896009.
E-mail: sales@psanalytical.demon.co.uk
Website: www.psanalytical.demon.co.uk

A digital photographic image is available on request (see below).

*Please contact Avril Stockwell at afs@psanalytical.demon.co.uk; Tel (Int): +44 (0) 1689 891211 Fax (Int) +44 (0) 1689 896009*

### Trade marks approved for P S Analytical

Over the past few months, P S Analytical has received approval for trade marks for the Merlin, Excalibur and Sir Galahad instruments. The style and presentation of these trademarks are set out below. Due to the popularity of these names, the trademarks include other symbols associated with the PSA product line.

The P S Analytical Merlin atomic fluorescence detector was the world's first full automatic mercury analyser. The PSA Excalibur extended the range of analytes to the hydride-forming elements, specifically arsenic, selenium and antimony.

The recently introduced Millennium range of products are specifically designed systems which provide routine analytical measurements for mercury, arsenic, selenium and antimony at ultra-trace levels, and offer a viable measurement tool for these analytes between 0.1 ppt for mercury and 5 ppt for the hydride-forming elements.

The Sir Galahad II has been specifically developed to anlyse mercury in a variety of gaseous environments, including air and natural gas. P S Analytical has earned a world-wide reputation for performing the latter application, particularly in difficult samples and sites, e.g. Natural Gas platforms. Recently, a new pressure let-down system has also been introduced which overcomes many of the inherent problems associated with sampling natural gas at high pressure in a truly representative manner.

These trademarks will further cement the reputation of P S Analytical's products which have followed the Arthurian theme since the introduction of the Merlin Detector in 1987.

*For further information on these products or others in the P A Analytical range, please contact: Paul Stockwell, P S Analytical, Arthur House, Crayfields Industrial Estate, Main Road, Orpington, Kent, BR5 3HP, UK. Tel (Int): +44 (0)1689 891211; Fax (Int): +44 (0)1689 896009; e-mail: sales@psanalytical.demon.co.uk;*
*Website: www.psanalytical.demon.co.uk*

### P S Analytical launches website

P S Analytical has recently launched its own website, provide instant access to information on the new Millennium Systems for the determination of mercury, arsenic, selenium, bismuth, tin in industrial and environmental samples, and also the Sir Galahad Mk II instrument for mercury determinations in gaseous samples.

The aim is to allow visitors to the site to review the products and services available from P S Analytical and to provide easy access to promotional and applications material.

The highly successful Millennium range of products, the Millennium Merlin and Millennium Excalibur, are self-contained systems which provide the sample preparation and transfer to specific atomic fluorescence detectors (AFS). The detectors offer significant improvements in detection capabilities for mercury, arsenic, selenium and antimony with outstanding reliability.

Surf the web and visit our site. Why not arrange a demonstration of a Millennium system, with the help of PSA and their worldwide distributor network, you will be pleasantly surprised at how easy they are to set up and achieve the detection levels quoted. Don't settle for less – what PSA claim is what you can readily achieve.

For further information on these products or others in the P S Analytical range visit the Website at www.psanalytical.demon.co.uk or contact Paul Stockwell at: P S Analytical, Arthur House, Crayfields Industrial Estate, Main Road, Orpington, Kent BR5 3HP, UK. Tel (Int): +44 (0)1689 891211; Fax (Int): +44 (0)1689 896009; E-mail: sales@psanalytical.demon.co.uk.

### New Shimadzu EZTest: Small on size, big on performance

Shimadzu EZTest is a new, small, universal testing instrument for a variety of laboratory research and control applications, including food, cosmetics, electronics, pharmaceuticals and packaging.

Although compact in size, the instrument is big on performance. It meets all DIN Class 1 measuring requirements and delivers accuracies to 1% with forces of 2 N and testing velocities of between 0.5 and 500 mm/min.

The application range of the EZTest is very wide – including food processing, packaging, pharmaceuticals, cosmetics and electronics – and it is suitable both as a stand-alone instrument for occasional testing and as a support system for testing laboratories with larger instruments who do not want to constantly readjust their main analyser for small force measurements.

Pull-, pressure-, three- and four-point stretch tests and adhesion tests can all be handled by the new instrument, and a number of different tensile strength tools are also available for specific applications.
Simple to use, EZTest is designed for one-button operation and is equipped with built-in programmable control.

| *Industrial area* | *Application* |
|---|---|
| Foods | Fruits, bread, chewing gum (*elasticity, chewing properties*) |
| Food packaging | Bottles, can openers (*tensile force, adhesion, pressure tests*) |
| Pharmaceuticals | Tablets, injection needles, infusion containers (*pressure tests, penetration force*) |
| Cosmetics | Soaps, lipsticks (*hardening properties, malleability*) |
| Electronics | Switches, integrated circuits (*tensile force*) |
| Packaging (general) | Foils, paper, cartons (*pressure tests, tear resistance*) |
| Adhesives | Adhesive labels, tape (*adhesive strength*) |

There is a printer port for production of documentation and the instrument can also be connected to a computer to gether testing protocols, statistics, etc. and linked directly to MS-ACCESS.

A clear arrangement of all screens is provided by WinAGS LITE software running on Windows 95. Optical help functions are available at all stages, displaying non-conventional sample geometry and, via icons, mode of operation. All graphics are displayed in real-time.

Each newly developed method is automatically entered into a database for later recall as needed. All routine parameters can be pre-determined for data evaluation and recalled after new data acquisition during testing, and the software includes a statistical package for re-analysis.

'The EZTest is good news for customers who are reluctant to purchase a large testing instrument', said Shimadzu's Brian Miller.

'We have taken some of the best innovations to come out of miniaturisation – now a major trend in materials testing – and incorporated them in a simple-to-use, highly effective universal testing instrument which has a multitude of industrial and research applications'.

For more information, please contact:
Sales enquires: Shimadzu Europa (UK Branch), Mill Court, Featherstone Road, Wolverton Mill South, Milton Keynes MK12 5RE, UK. Tel: (+44) (0)1908 552200; Fax: (+44) (0)1908 552211; e-mail: Sales@Shimadzu.co.uk.
Press enquiries: Ross Heaven, Strong Words, 32 Cranstoun Street, Northampton NN1 3BH, UK; Tel/Fax: (+44) (0)1604 250221.

### New Shimadzu DSC-60: The start of a comprehensive new TA line

Building on its experience as the first manufacturer of thermal analysis instrumentation, Shimadzu has developed the DSC-60 differential scanning calorimeter, a new analyser within the popular TA-60 thermal analysis range.

The DSC-60 offers high sensitivity, low noise levels and excellent signal resolution, all within a compact, modern design, which also features an integrated cooling system and can be equipped with an autosampler for routine analyses in food, ceramics, petrochemical, plastics, adhesives, pharmaceutical, coatings, environmental and other typical application areas.

The system runs on TA-60WS software, a 32-bit Windows-based enhancement of the TA-50WS package, which has been designed for greater user-friendliness and easy maintenance.

Automatic evaluation algorithms have been incorporated to simplify routine analyses and smoothing functions, baseline corrections and calibration can all be pre-set according to specific laboratory methods, creating a one-button system for instrument operation.

'It is our intention to develop, enhance and improve the TA-60 line to ensure that Shimadzu—the pioneers of thermal analysis—continue to stay at the forefront of this analytical area', said Brian Miller of Shimadzu.

'By the year 2000, the TA-60 series will feature a complete instrument line of DSC, TGA, simultaneous TA, DMA and TMA instruments for temperatures of −150°C to 1500°C. Customers can visit our web site or e-mail at sales@shimadzu.co.uk for further information on these developments as they take place'.

For more information, please contact:
Sales enquires: Shimadzu Europa (UK Branch), Mill Court, Featherstone Road, Wolverton Mill South, Milton Keynes MK12 5RE, UK. Tel: (+44) (0)1908 552200; Fax: (+44) (0)1908 552211; e-mail: Sales@Shimadzu.co.uk.
Press enquiries: Ross Heaven, Strong Words, 32 Cranstoun Street, Northampton NN1 3BH, UK; Tel/Fax: (+44) (0)1604 250221.

### Shimadzu Micro-system offers new analytical benefits

A powerful new micro- and capillary option for the LC-10A and LC-10A VP HPLC Series has been launched by Shimadzu in response to the growing number of chromatographic applications using columns with an internal diameter of less than 1 mm, from areas. e.g. biotechnology.

By upgrading a conventional HPLC system to a Micro-Workstation, users benefit from a raft of new advantages, including highly efficient handling of small sample volumes, flow rates from 50 nl/min to 10 ml/min, fast equilibration characteristics for fast gradients, superior RSD values, better UV-sensitivity (noise <30 μAU), and flexible use of DAD and fluorescence detector, all with greater user-friendliness at an attractive price.

One further, very powerful benefit of the Shimadzu Micro-Workstation is its gradient reproducibility, which is currently unsurpassed by any other micro-system.

'The move towards capillary HPLC has resulted from the need to decrease solvent consumption and to handle smaller samples more effectively', said Brian Miller of Shimadzu.

'Capillary LC, routinely used in a variety of applications, is the method of choice where small samples volumes are available—such as single bead analysis in combinatorial chemistry, spot analysis following 2D-electrophoresis, and water trace analysis in the environmental field'.

'With the new Shimadzu Micro-Workstation, the analyst now has access to high sensitivity and resolution with the best gradient reproducibility currently available'.

For more information, please contact:
Sales enquires: Shimadzu Europa (UK Branch), Mill Court, Featherstone Road, Wolverton Mill South, Milton Keynes MK12 5RE, UK. Tel: (+44) (0)1908 552200; Fax: (+44) (0)1908 552211; e-mail: Sales@Shimadzu.co.uk.
Press enquiries: Ross Heaven, Strong Words, 32 Cranstoun Street, Northampton NN1 3BH, UK; Tel/Fax: (+44) (0)1604 250221.

### PAAR sorts out the 'jellification' point

Producers of high value fruit compote from apricots, strawberries, plums and raisins, etc. with narrow viscosity specifications, often involuntarily deliver juice concentrate. 'Viscosity beyond customer's specification' is usually given as a reason.

The pectin content and the proportion of fruit water to fruit and sugar content are all factors in determining the 'jellification' point of the fruit compote. The fruits show no consistent characteristics, due to different factor, e.g. location, hours of sun, ripeness and weather during

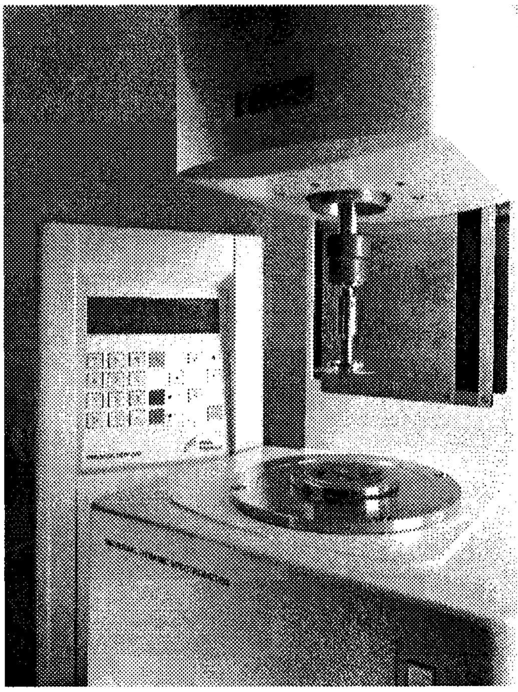

*UDS 200 Universal Dynamic Spectrometer.*

harvest time, which varies due to a Europe-wide procurement basis.

Ingredients that influence the jellification point are highly concentrated and expensive, requiring exact proportioning. Even small mistakes in amount can have a lasting impact on the viscosity of the fruit jelly. Once batches of 1000–10 000 kg are produced and cooled down, the viscosity of the fruit jelly has to be as originally specified, as corrections cannot be later rectified.

PAAR has now introduced a pressure-measuring cell, combined with a UDS200 rheometer, designed specifically to determine almost all rheological data relevant for practice and research. The PAAR cell accounts for the raw food material under processing conditions by simulating production pressures of up to 6 bar and temperatures from −10 °C to +150 °C.

The data determine the jellification point of the fruit compote before production starts, so loss of taste of the fruit content due to over-processing or too high temperature is virtually eliminated.

PAAR can therefore meet the demands of producers, e.g. manufacturers of ice cream, who insist only the correct quality of fruit concentrate is supplied to them, otherwise the fruit goes to soft drinks manufacturers, where the price of supply is lower.

*For further information and reader enquiries, contact Mr Bryan Treherne at PAAR Scientific, 594 Kingston Road, Raynes Park, London SW20 8DN, UK. Tel: +44 (0)181 540 8553. Fax: +44 (0)181 543 8727. E-mail: paar@psl.anton-paar.co.uk*

### Perkin-Elmer's Tropix subsidiary implements new Zymark automation system

*System expected to provide capacity of 400 000 assays daily for drug discovery screening services*

*Business wire—15 June 1998; Bedford, MA (BW Healthwire)—15 June 1998:* Tropix, a subsidiary of Perkin-Elmer (NYSE: PKN), and Zymark Corporation are installing Zymark's new 'Allegro' automation technology at the Tropix Advanced Discovery Services laboratory here. Tropix's Xtreme Screen program offers pharmaceutical customers high-throughput drug discovery screening services employing high-performance assays using the company's advanced, proprietary chemiluminescent technology.

According to Tropix President Dr Irena Bronstein, 'The "Allegro" automation system will be used as a "rapidly re-configurable assay machine" initially capable of delivering over 100 000 assays per day under the demanding extreme usage conditions.'

The system, which uses robust, proven technology, consists of inter-linked modular workstations in a unique assembly-line architecture. With advanced technology from both organizations, the capacity of the 'Allegro' system is expected to be expanded to 400 000 assays per day. Ultimately, the Allegro automation engine may be coupled to a broad range of Perkin-Elmer proprietary technology platforms including RNA quantitation, mass spectrometry, static cytometry and ultra-miniature assays.

Kevin Hrusovsky, Zymark's president and chief executive officer, said, 'We're very happy to collaborate with Tropix. The company asked us for an ideal platform to incorporate future innovations in their assay chemistry and a system that can be easily and rapidly reconfigured to meet their customers' changing needs while assuring future compatibility with advances in liquid handling and emerging low volume assay technology. We are very pleased that Tropix has joined the ranks of other "Allegro" development collaborators such as R. W. Johnson Pharmaceutical Research Institute and Boehringer Ingelheim Pharmaceuticals. "Allegro" will provide the throughput, reliability, and flexibility to meet the demanding challenges of their customers' drug discovery requirements.'

Zymark Corportion, headquartered in Hopkinton, MA, is a worldwide leader in laboratory automation for pharmaceutical and biotechnology applications. Zymark serves the worldwide pharmaceutical and biotechnology industries. The company employs about 300 people in the USA, Canada and Europe, and has authorized sales and service distributors throughout the rest of the world.

Tropix, located in Bedford, MA, is a world leader in the development, manufacture and sale of chemiluminescence detection technology for the life sciences. The company's Xtreme Screen program provides a three-tiered approach to delivering advanced chemiluminescent screening technologies. First, it provides proprietary chemiluminescent dioxetane screening reagents to meet the stringent demands of yielding high quality data across a wide range of assay types in a single platform at extreme throughput levels in a cost-effective manner. Second, it rapidly develops custom screening assays for deployment on screening systems at the user's site. Third, it is a complete service to perform advanced screens at rates above 100 000 samples per day with in-house or customer-supplied libraries, bringing extremely rapid turnaround on large or small screening projects. The combination of the three elements significantly contributes to accelerating the drug discovery timelines of pharmaceutical company clients. The Perkin-Elmer Corporation is a leading supplier of systems for life science research and related applications. It develops, manufactures and markets life science systems and analytical instruments used in markets, such as pharmaceutical, biotechnology, environmental testing, food, agriculture and chemical manufacturing. Headquartered in Connecticut, Perkin-Elmer had revenues of nearly $1.4 billion in fiscal 1997 and employs more than 6000 people worldwide. Information about Tropix is available on the World Wide Web at http://www.tropix.com. Information about Perkin-Elmer is available on the World Wide Web at http://www.perkin-elmer.com or by phoning (800) 762-6923.

Certain statements in this press release are forward-looking, and are subject to a variety of risks and uncertainties. These statements may be identified by the use of forward-looking words or phrases, e.g. 'believe', 'expect', 'anticipate', 'should', 'planned', 'estimated' and 'potential', among others. These forwardlooking statements are based upon Perkin-Elmer's current expectations. The Private Securities Litigation Reform Act of 1995 provides a 'safe harbour' for such forward-looking statements. In order to comply with the terms of the safe harbour, Perkin-Elmer notes that a variety of factors could cause actual results and experience to differ materially from the anticipated results or other expectations expressed in such forward-looking statements. The risks and uncertainties that may affect the operations, performance, development and results of Perkin-Elmer businesses include, but are not limited to: (i) complexity and uncertainty regarding the development of new high-technology products; (ii) loss of market share through competition; (iii) introduction of competing products or technologies by other companies; (iv) pricing pressures from competitors and/or customers; (v) changes in the life sciences or analytical instrument industries; (vi) changes in the pharmaceutical, environmental, research or chemical markets; (vii) dependence on customers' capital spending policies; (viii) variable government funding in key geographical regions and its effect on government sponsored resarch; (ix) Perkin-Elmer's ability to protect proprietary information and technology, or to obtain necessary licences on commercially reasonable terms; (x) the loss of key employees; (xi) fluctuations in foreign currency exchange rates; and (xii) other factors which might be described from time to time in Perkin-Elmer's filings with the Securities and Exchange Commission.

*For further information, contact Perkin-Elmer, Norwalk, CT, USA. Investors: Charles Poole, 203/761-5400. Media: Edward Bloch, 203/761-5248. Zymark, Hopkinton, MA, USA. Media: Sharon Correia, 508-497-6403. sharon.correia@zymark.com*

### Perkin-Elmer's Tropix laboratory performs record number of pharmaceutical screening assays

*96 000 complex, high-throughput tests completed in 24 h period*

*Business wire—27 August 1998 08:45; Bedford MA—(BW Health wire)—27 August 1998:* Tropix, a subsidiary of Perkin-Elmer (NYSE: PKN) and a centre of excellence of its PE Biosystems Division, has validated its Allegro™ automation platform from Zymark Corporation by performing 96 000 pharmaceutical screening assays within a 24 h period. During a single day, Tropix processed a record 1000 96-well format microplates performing an advanced 15-step assay designed to screen for pharmaceutical candidates that inhibit src kinase activity. Src is an important kinase implicated in oncogenesis events related to certain cancers. According to Dr Irena Bronstein, president of Tropix, 'We believe this is the first practical demonstration of what has been termed "ultra high-throughput screening". Many organizations engaged in drug discovery work have talked about performing pharmaceutical screening assays at this level of throughput, but only Tropix has delivered. In addition, we performed the work with real-time data acquisition and analysis'. Dr Michelle Palmer, Tropix's director of pharmaceutical services, said 'Performing this number of kinase assays in 24 hours would have previously required nearly 15 conventional high-throughput robotic screening systems, or taken 15 times as long. The throughput capability we possess enables us to perform screens extremely quickly, providing our customers with many benefits beyond speed. One such benefit is maintaining assay performance during a screen which requires reagents that are only stable for short periods of time'. Tropix expects to further increase its laboratory throughput when the Allegro system is expanded to handle up to 400 000 assays per day. The src kinase assay in this screen was designed under Tropix's Xtreme Screen™ program, utilizing the company's proprietary adamantyl- dioxetane luminescence technology, which enables detection of biological substances at extremely low levels. The Xtreme Screen™ program offers pharmaceutical customers high-throughput drug discovery-screening services employing high-performance assays using the company's technology. The sensitive kinase assays performed to reach this milestone were measured using two robotics-capable Tropix TR717™ microplate luminometers integrated into the Allegro™ automation platform.

Zymark Corporation, based in Hopkinton, Massachusetts, developed the Allegro™ automation platform as part of a collaboration with Tropix. Kevin Hrusovsky, Zymark's president and chief executive officer, said, 'We're very proud to have played a role in Tropix's achievement of a thousand plates in one day. The Allegro™ platform is one of several key technologies we have developed over the past 18 months to fundamentally boost the speed and enhance the effectiveness of drug discovery. The quick success of the tropix installation reinforces Zymark's commitment to deliver "real results now" '.

### New brochure on Thermo Jarrell Ash's Atomscan Advantage series spectrometer

*Franklin, MA—August 1998:* Thermo Jarrell Ash Corporation (Franklin, MA) has available a new four colour, eight page brochure on their new sequential ICP spectrometers. The new Atomscan Advantage employs an innovative, high-performance optical design. A high-density composite grating delivers high resolution and performance over the entire wavelength range (160–900 nm). Two detectors with different spectral ranges are optimized to provide maximum sensitivity for both low UV elements (e.g. Al at 167 nm) and near IR elements (K at 766 nm). At the heart of the optical design is TJA's exclusive galvanometer grating drive that provides speed, accuracy and the ultimate in reliability. The galvanometer grating drive has been utilized in all Thermo Jarrell Ash sequential instruments for over 20 years, proving itself in millions of elemental determinations. The positioning accuracy of the galvanometer drive will never deteriorate with use, no matter what the demands.

The Atomscan Advantage may be configured as an axial or radial instrument. The radial configuration is preferred when analysing a wide variety of sample types due to the freedom from matrix effects. Axial viewing provides the ultimate in sensitivity when detection limit performance is key.

The Atomscan Advantage operates using ThermoSPEC®/PMT software, designed to fully control the analytical process without fully controlling the operator. All aspects of torch operation, including nebulizer pressure, auxiliary argon flow and RF power are controlled by the host software to ensure reproducible results. It is designed to run using the familiar Windows 98 operating system with a common look and feel, and includes the capability to export data to any popular database manager or spreadsheet program.

For a copy of the new Atomscan Advantage brochure, or any of Thermo Jarrell Ash's products, please call or write to Thermo Jarrell Ash, 27 Forge Parkway, Franklin, MA 02038, USA. Tel: +1 (508) 553-1200, Fax: +1 (508) 520-1732, or visit our web site at http://www.tja.com/~tjabaird/

### New brochure on Thermo Jarrell Ash's IRIS DCP spectrometer

*Franklin, MA—August 1998:* Thermo Jarrell Ash Corporation (Franklin, MA) has available, a new four colour, six page brochure on their new simultaneous plasma emission spectrometer which features a direct current plasma source coupled to the CID-echelle spectrograph. The new IRIS DCP provides exceptional performance in a low-cost, benchtop instrument.

The IRIS DCP utilizes the Spectrajet III, a plasma emission source which is renowned for simplicity and reliability. The sample introduction system employs a ceramic nebulizer, polypropylene spray chamber, PVC aerosol transfer tubing, and a graphite aerosol introduction tube. The absence of silicon-containing materials simplifies analysis of HF solutions: no special components, tedious alignment, or extra-complex sample preparation is required. At the same time, the sample introduction system readily handles dissolved solids levels of 30% or more, minimizing loss of sensitivity due to sample dilution.

The IRIS DCP utilizes the charge injection device array detector coupled to high-resolution echelle optics. The operator may choose from a virtually unlimited number of analytical lines between 184 and 900 nm to minimize interferences as well as to obtain required sensitivity for each sample matrix. Because the CID array measures lines simultaneously, there is no increase in analysis time. And, there is a signal-to-noise advantage from simultaneous measurement of background. The instrument is controlled by ThermoSPEC®/WIN™, a comprehensive software package that is multitasking in Windows™, so that the operator may utilize spreadsheets, word processors, etc. while ThermoSPEC/WIN continues to control the instrument and collect data.

For a copy of the new IRIS DCP brochure, or any of Thermo Jarrell Ash's products, please call or write to Thermo Jarrell Ash, 27 Forge Parkway, Franklin, MA 02038, USA. Tel: +1 (508) 553-1200, Fax: +1 (508) 520-1732, or visit our web site at http://www.tja.com

### New water sorption analyser automates complex test sequences

Salisbury-based C.I. Electronics launches an innovative water sorption analyser which automates gravimetric study of the water sorption behaviour of powdered, fibrous, liquid or solid samples to quickly and economically provide precise results in the chemical industry.

Based on microbalance technology developed by a world leader in its field, the CISorp Water Sorption Analyser represents a major advance over previous analytical methods. In contrast to established techniques which are typically slow, labour intensive, and potentially inaccurate, CISorp allows unattended execution of complex test sequences with high accuracy and dramatically improved productivity.

CISorp measures moisture adsorption and desorption isotherms, plus kinetic and temperature data for evaluation of important physical and chemical parameters in research and development, quality control, and determination of safe storage and handling conditions for materials. Operating at ambient pressures, CISorp is particularly suited to the study of hydrated samples which generate high vapour pressure and cannot be studied under vacuum.

Water sorption studies can reveal crucial information about samples which may not be obtained in any other way, and highlight subtle differences between compounds which may escape other analytical techniques.

With its flexibility of operation, CISorp yields information, e.g. the general water sorption characteristics of a sample, the physical and chemical affinity of sample surfaces for water, the presence of surface pores, irreversible change resulting from water sorption, special features, e.g. transitions at particular humidities, variations between samples and the effects of mixing them, the effect of compacting powders, the temperature dependence of water sorption, and the thermodynamic and kinetics quantities of water sorption. CISorp incorporates two microbalances in a chamber in which temperature can be controlled in the range 5–65°C with ±0.1°C accuracy, and relative humidity (RH) in the range 0–100% with 0.1% resolution. A test sequence can be programmed with a large number of experimental steps, at which temperature, humidity or equilibration time may be specified, and the weight of the samples measured with 0.1 µg resolution. The dual balance arrangement enables simultaneous analysis of two samples or comparison of a sample against a standard.

PC Windows-based software controls data acquisition, display and printout of results. Tabular and graphical displays can be viewed in real-time, and recalled for review or printout after a test.

For further information contact Elaine Roberts, C.I. Electronics Limits, Brunel Road, Churchfields, Salisbury, Wiltshire SP2 7PX. Telephone: +44 (0)1722 336938. Fax: +44 (0)1722 323222. email: admin@cielec.com or discover CISorp at www.cielec.com.

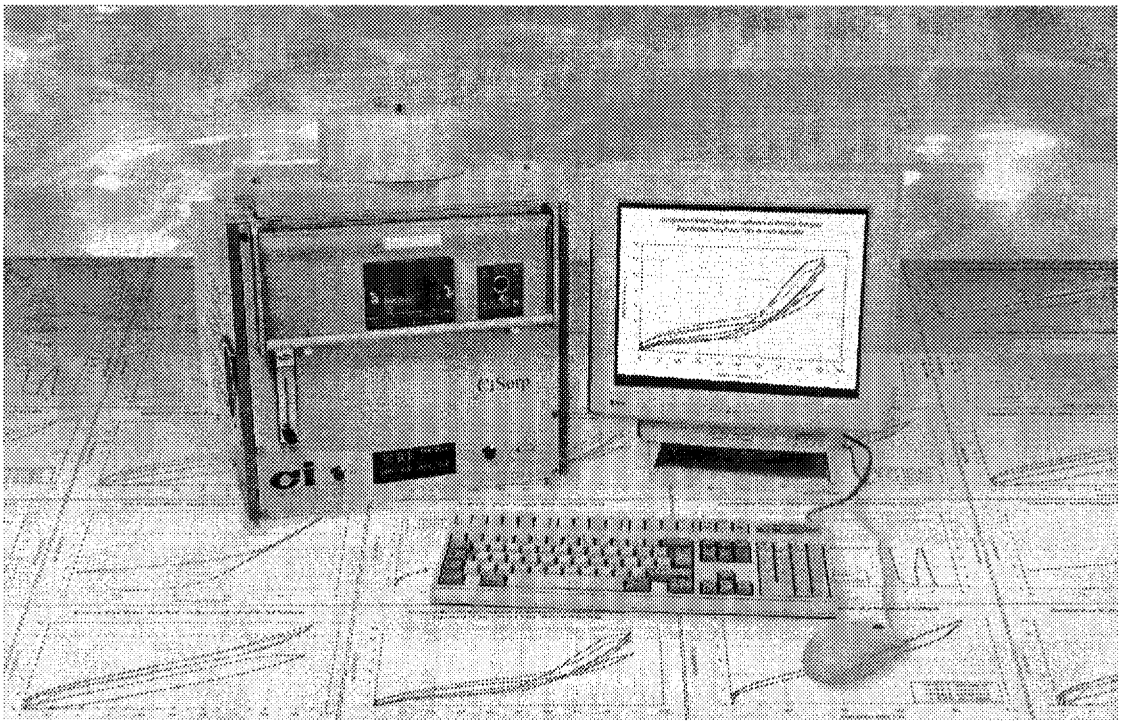

*The innovative CISorp water sorption analyser from C.I. Electronics automates measurement of parameters crucial to characterisation of materials.*

### New Hydrogen Mate produces high purity deionized water economically for Whatman hydrogen generators at point of use

A new economical means of producing deionized water for hydrogen generators, as now available from Whatman International.

It is called the Hydrogen Mate DI Water System which has been designed for the sole purpose of economically producing high-purity deionized water for use with all Whatman hydrogen generators.

The unit will remove organics, phosphates, chlorine and essentially all ionizable constituents from the water supply. It has an easy-fill dispensing gun and there is a visual indication for cartridge replacement.

The Hydrogen Mate is complete and ready to install, and simply operates by piping into the laboratory water supply not exceeding 34.5 bar. It achieves a maximum flow rate of 1 l/min.

The compact unit weights only 5 kg, and its dimensions are 311 wide × 457 high × 73 mm deep.

### Building blocks for chemistry education on the Web

Berlin, 24 July 1988—The Fachinformationszentrum CHEMIE GmbH Berlin (Fiz Chemie Berlin, The Chemistry Information Centre) won a national competition held by the *Federal German Ministry of Education, Science, Research and Technology* (Bundesministerium für Bildung, Wissenschaft, Forschung und Technologie, BMBF) for the formulation of new research projects concerned with the use and application of world-wide information for education, further education and innovative processes.

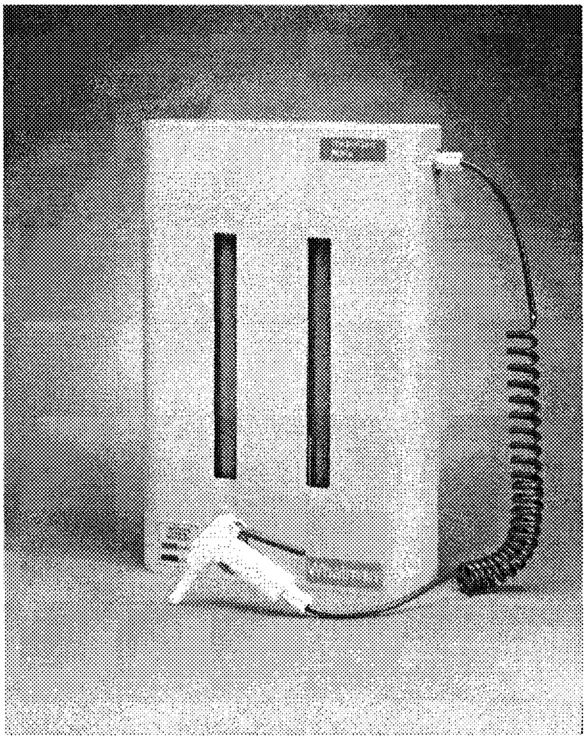

*The Whatmann Hydrogen Mate.*

Of the 251 applications submitted, five winners were selected by the BMBF based on their innovative content, including the Fiz Chemie Berlin project 'Vernetztes Studium—Chemie' ('Chemistry Networks for Education'), which describes a new form of studies in chemistry. The five winning projects will be supported financially by the BMBF for the next five years.

The 'Chemistry Networks for Education' project foresees the development of interactive 'knowledge building blocks' and information tools largely based on multimedia technology, which will then be made available both via the Internet and as an in-house version. The material itself will be produced in the form of so-called 'knowledge modules'.

In all, 16 professors from 13 German universities located in eight of the Federal German states will be working on the project under the expert guidance of Fiz Chemie Berlin. The ultimate goal is to create an electronic platform which will, in optimized networked form, contain all knowledge in chemistry available world-wide and will be accessible by those outside traditional campus environments. Thus, because of the modular character of the information on offer, it will not only be well-suited to the needs of university under- and postgraduates in chemistry and its related sciences, but also to students at polytechnics and colleges of further education, for job trainees and school students. Even the professional or private information requirements of persons not directly concerned with chemistry will be met by the system.

The concept of 'Chemistry Networks for Education' also encompasses a model proposed by the German Chemical Society (Gesellschaft Deutscher Chemiker, GDCh) in conjunction with the German universities for a reformation of university chemistry education in Germany, which was proposed in a joint memorandum in June 1996 as the so-called 'Würzburger Model'.

The project aims at improving education in chemistry by making use of all modern methods of transmitting information. Designed to supplement and support conventional forms of study, it thus employs electronic media to rapidly integrate new material and information into study courses. It also facilitates the use of up-to-date information available from databases and employs novel presentation forms, e.g. participation in interactive lectures mediated via the Internet or the carrying out of 'experiments' in virtual laboratories.

These novel forms of teaching and learning are expected to result in both a reduction in the lengths of time required for studies in chemistry and to have positive effects on the European harmonization of education in chemistry as a whole.

For further information, please contact: Fiz Chemie Berlin, Franklinstrasse 11, D-10587 Berlin, Germany or Fiz Chemie Berlin, Postfach 12 03 37, D-10593 Berlin, Germany. G. Brzoskniewicz, Telephone: 030/399 77-250; Telefax: 030/399 77-135; E-mail: brzos@fiz-chemie.de; Internet/Web-site: http://www.fiz-chemie.de: E.mail: info@fiz-chemie.de

### Evaluation of drug discovery/development systems from Micromass

In 1998, Micromass introduced a new family of LC-MS workstations featuring *ZSPRAY*™ ion source technology and on-line LC-MS exact mass measurement capability. Optimized for intensively automated pharmacokinetic studies, *ZSPRAY*™ fully satisfies the pharmaceutical industry's criteria for a robust API LC-MS interface with uncompromised sensitivity. Furthermore, its easily accessible open architecture simplifies the system's operation with NanoFlow ES—the analytical technique preferred by proteome investigators in the bio-pharmaceutical sector.

This year Micromass' proven orthogonal acceleration time-of-flight technology has been refined into a bench-top drug discovery workstation with the LCT™ 'Walk-Up' Exact Mass LC-MS. The new system can be configured for 'open access' LC-MS with the power of exact mass measurement to provide chemists with elemental composition data.

Also new for 1998 is an expanded range of autopurification/fraction collection systems with greatly enhanced sample loading capacity and flexibility, known as FractionLynx™. Quattro LC is Z*SPRAY*™ enabled for 1998; the pharmaceutical industry's triple quadrupole has been enhanced with Micromass' quantum leap in API ion source technology Z*SPRAY*™, two orthogonal ion sampling zones delivering double the power—unmatched robustness and sensitivity.

Q-Tof™ continues to dominate new analytical challenges. Micromass' paradigm shift in LC-MS-MS now addresses key emerging pharmaceutical applications (in addition to proteomics): low level metabolite identification, deconvolution of complex combinatorial libraries and patent protection.

As a detailed proof statement of Micromass' work in areas from drug discovery to end product, an application note (no. 233) has been published to evaluate the Z*SPRAY*™ API ion source for high throughput analysis of drugs directly from cell culture media at high ionic strength. Written in conjunction with members of the Exploratory Drug Delivery Group from Pfizer Central Research, the application note assesses the potential of a novel, dual orthogonal sampling, API ion source for the direct analysis of drugs in (un-desalted) cell culture media of the type typically employed in *in vitro* drug absorption studies.

The report states, "Contrary to our experiences with other mass spectrometer sources, the Z*SPRAY*™ source is entirely suitable for the analysis of samples with high electrolyte concentrations. Indeed, we anticipate that Z*SPRAY*™ source technology will prove advantageous applied to the analysis of other 'dirty' samples such as protein-precipitated plasma, urine, bile, and extracts from a variety of cell culture experiments."

If you would like this test e-mailed directly to you, contact Strategic p.r. on helen.samuel@bath-gb.demon.co.uk; or supplied on disk, fax requirements to +44 (0)1225 480669.

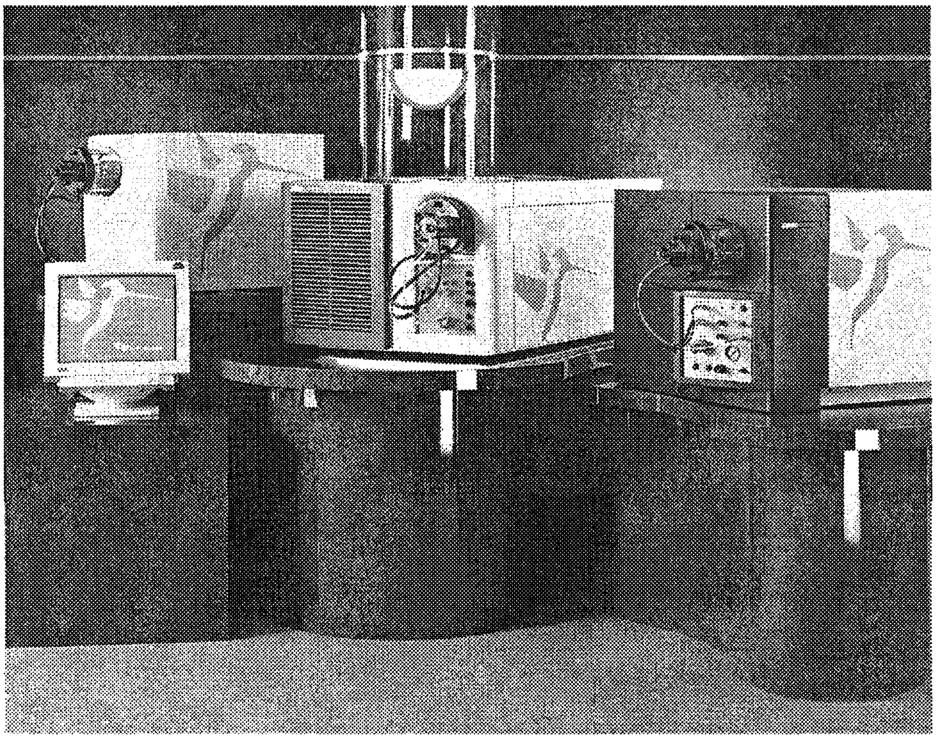

*Micromass ZSPRAY™ source available on a range of Micromass mass spectrometers.*

For further information contact: Editorial contact: Helen Samuel, Strategic p.r., 38 Combe Park, Weston, Bath BA1 3NR, UK. Tel: +44 (0)1225 480667. Fax: +44 (0)1225 480669. E-mail: helen.samuel@bath-gb.demon.co.uk

Lead fulfilment (Europe): Micromass UK Limited, Response Handling (IAS), Queens Avenue, Macclesfield, Cheshire SK10 2BN, UK. Tel: +44 (0)1625 434343. Fax@ +44 (0)1625 434335.

Lead fulfilment (USA): Joanne Libert, Micromass Inc., 100 Cummings Center, Suite 407 N, Beverly, MA 01915-6101, USA. Tel: + +1 (978) 524 8306. Fax: + +1 (978) 524 8210. E-mail: joanne_libert@mspeople.com

Company contact: Steve Preece, Micromass UK Limited, Tudor Road, Altrincham, Cheshire WA14 5RZ, UK. Tel: +44 (0)161 282 966. Fax: +44 (0)161 282 440. E-mail: steve.preece@micromass.co.uk

### New developments in *de novo* peptide sequencing by ESI-MS-MS

In its new literature, 'Towards Automated *De Novo* Peptide Sequencing by ESI-MS-MS' (Application Note No. AN234/ICA Version 1), Micromass gives an account of research conducted to show that *de novo* sequences may be determined from MS-MS spectra by automated software interpretation.

With the size increase of protein and EST databases, and genomes becoming more completely sequenced, more and more protein identification will involve peptide fingerprinting of the products of enzymatic digestion, or partial, ESI-MS-MS-derived sequences (tags). However, organisms with incomplete genomes, or with poorly populated protein and EST databases, will require substantial protein sequence determination.

Automated Edman remains the dominant protein sequencing method. Recent new developments in mass spectrometry, though, have produced an exciting alternative. The current technique involves digesting and 'tagging' the protein, and, from the resulting ($^{18}O$) tagged peptides, producing MS-MS data. However, whereas the Edman technique produces a single sequence from the N terminal end, MS-MS of peptides almost always results in sequence information from both the N and C terminal end of the molecule within the same spectrum. Data interpretation is, therefore, more complicated since these opposing ion series must be disentangled. however, when rationalized, the complementary N and C terminal ion series can afford a 'double confirmation' of amino acid sequence.

The new automated MS-MS procedure described in this publication alleviates the complexity of disentangling N and C terminal MS-MS sequence data. *De novo* sequence interpretation of MS-MS spectra may be facilitated by tagging ($^{18}O$) the peptide in a way which will identify the sequence ions containing the C terminal end of the molecule. If the digestion is carried out in a (1 : 1) mixture of $^{16}O/^{18}O$ enriched $H_2O$, endoproteinases will incorporate either a $^{16}OH$ or $^{18}OH$ into the peptide, resulting in a characteristic isotopic 'signature' in the mass spectra.

Achievable sensitivity has been increased by the recent design and construction of a hybrid quadrupole time-of-flight (Q-Tof) tandem mass spectrometer, the advent of which has also seen improved resolution and mass meas-

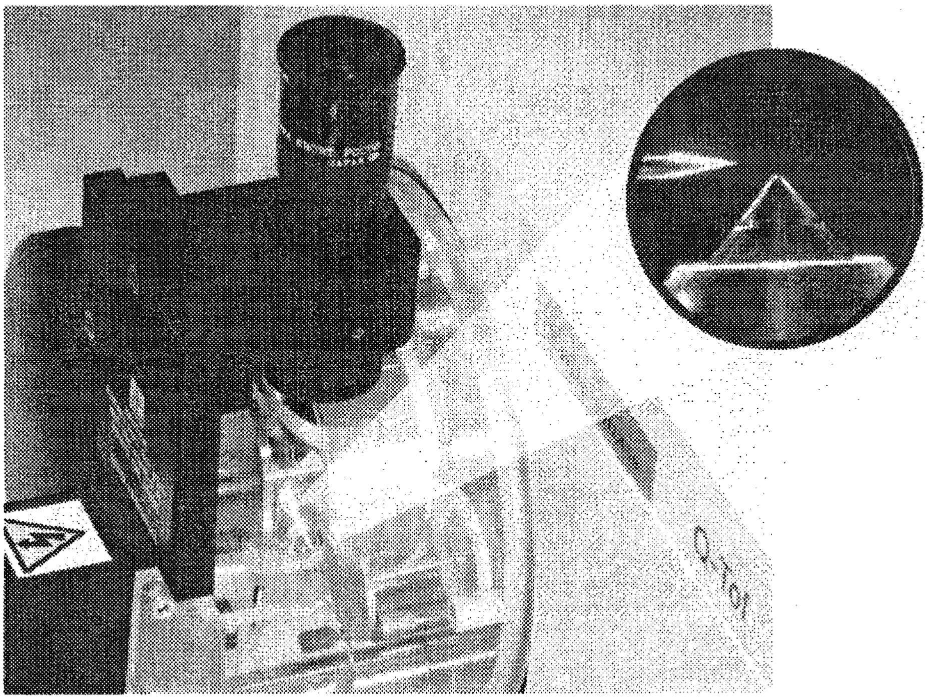

*Micromass hybrid quadrupole time-of-flight (Q-Tof) tandem mass spectrometer.*

urement accuracy for MS-MS data. When used in conjunction with nanoelectrospray ionization, high quality sequencing data from in-gel digested 1D bands and 2D spots is readily produced. Furthermore, computer software may be used to search the spectra for these 1 : 1 $^{16}O/^{18}O$ multiplets and automatically infer amino acid sequences.

Having already proved itself a major competitor in the field of proteomics, Micromass' latest efforts to improve and facilitate high throughput/automated peptide sequencing techniques is further evidence of its all embracing biopharmaceutical strategy—across the continuum from DNA to drug product!

If you would like the text e-mailed directly to you, contact Strategic p.r. on helen.samuel@bath-gb.demon.co.uk, or supplied on disk, fax requirements to + 44 (0)1225 480669.

For further information contact:

Editorial contact: Helen Samuel, Strategic p.r., 38 Combe Park, Weston, Bath BA1 3NR, UK. Tel: +44 (0)1225 480667. Fax: +44 (0)1225 480669. E-mail: helen.samuel@bath-gb.demon.co.uk

Lead fulfilment (Europe): Micromass UK Limited, Response Handling (IAS), Queens Avenue, Macclesfield, Cheshire SK10 2BN, UK. Tel: +44 (0)1625 434343. Fax: +44 (0)1625 434335.

Lead fulfilment (USA): Joanne Libert, Micromass Inc., 100 Cummings Center, Suite 407 N, Beverly, MA 01915-6101, USA. Tel: + +1 (978) 524 8306. Fax: + +1 (978) 524 8210. E-mail: joanne_libert@mspeople.com

Company contact: Mark McDowall, Micromass UK Limited, Tudor Road, Altrincham, Cheshire WA14 4RZ, UK. Tel: +44 (0)161 282 9666. Fax: +44 (0)161 282 440. E-mail: mark.mcdowall@micromass.co.uk

### New bench-top density meter offers higher accuracy at lower cost

The latest product off the launch pad at PAAR Scientific is the DMA 4500 bench-top density meter for liquids and gases, offering high accuracy, fast measurement and a lower cost per measurement, plus it is easy to use and is highly versatile.

DMA 4500 is completely new with a combination of the latest developments in operating software, user interface and measuring cells. PAAR claim the instrument is more accuracy than any four-place digital density meter on the market, featuring an accuracy of 0.00005 g/cm$^3$, at an economical price.

The new software will meet the most demanding requirements. All common concentration tables including Brix, alcohol, acids and bases, are already integrated in the instrument to provide automatic concentration calculation. Even the API software is permanently stored, so that the density measured at any temperature from 0 to 90°C is automatically compensated to the reference temperature of 15°C or 60°F.

For uncommon density applications, the flexible software lets the user enter his/her own tables or formulae very easily using the instrument keys, or, even more conveniently using a PC keyboard. The keyboard connector can also be used to attach a bar code reader for safe and convenient entry of sample names and numbers.

The DMA 4500 features a reference oscillator for exceptional long-term stability and fast measuring results within 1–2 min. After changing the measuring tempera-

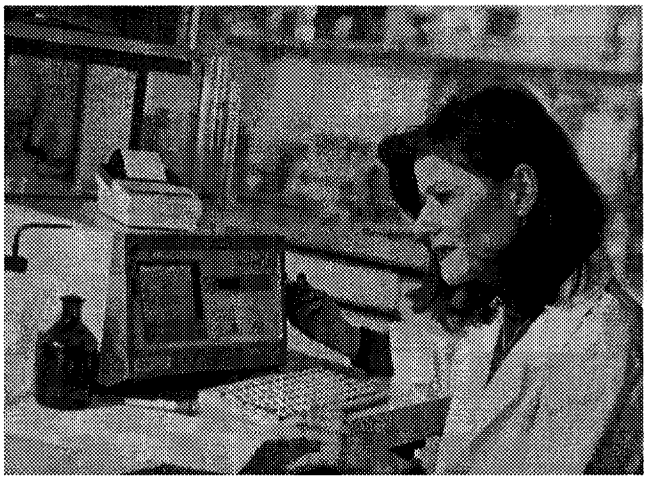

*PAAR Scientific new bench-top density meter.*

ture, accurate measurements can be performed immediately the target temperature is reached. The powerful solid-state thermostat handles temperatures from 0 to 90°C. A platinum temperature probe is built into the density sensor to provide accurate sample temperatures.

Viscosity-related errors inherent to all older models of density meters using the vibrating U-tube principle, are automatically eliminated by the new instrument. The viscosity influence is automatically corrected by determining the viscosity-related damping of the U-tube's oscillation. The DMA 4500 therefore meets even the most recent ASTM standards for the petroleum industry.

Serial interfaces for printer connection, PC or LIMS are standard features. Automatic sample chargers for fully automated sample filling and cleaning are useful options. PAAR believe the DMA 4500 will be a very successful instrument answering the needs of users now and into the next millennium.

For further information and reader enquiries: Bryan Treherne at PAAR Scientific Limited, 594 Kingston Road, Raynes Park, London SW20 8DN. Tel: 0181 540 8553; Fax: 0181 543 8727.

### New Metrohm 766 Ion Chromatography Sample Processor

The 766 Ion Chromatography Sample Processor from Metrohm is capable of handling sample volumes up to 11 µl, and thus compliments the Metrohm 750 Sample Charger who's maximum sample volume is 700 ml. The 127 sample of the 766 are arranged in three rows, which guarantees easy access and unrestricted programming. Two additional rinse positions allow sample introduction free from cross-contamination, even with a wide variety of sample matrices.

The sample vials are made of polypropylene and these can be heretically sealed. The needle on the 766IC Sample processor penetrates the cap of the sample vessel and the integrated pump conveys the sample through the sample loop to the injector. A sequence can be programmed such that an air bubble is inserted in front of the sample to prevent cross-contamination.

The 766IC Sample processor processes not only the normal sample series, but can also be used for sample enrichment with pre-columns, for operations involving the 754 Dialysis Unit as well as in a parallel system, e.g. for the simultaneous determination of anions and cations.

For further information please contact Metrohm UK, Tel. 01280 824 824.

### Auto self-cleaning dissolved oxygen monitor

Tried and tested in the USA for over seven years, and claimed to be up to 50% cheaper than its rivals, the first auto self-cleaning dissolved oxygen monitor has now been launched in the UK by ATi (UK) Ltd.

Their Auto-Clean $DO_2$ equipment is built to perform in the most demanding environments with minimal operator attention. The applications are many and varied including brewing, river monitoring and fish farming, chemical processing, paper and pulp production, together with sewage and industrial effluent treatment.

In operation, oxygen diffuses through a Teflon membrane in the galvanic sensor which triggers a small electrical current proportional to the amount of dissolved oxygen concentration. A water temperature measurement is also taken and is used to adjust the sensor signal. The result is a dissolved oxygen measurement that is accuracy over an operating range of 0–50°C.

The Auto-Clean $DO_2$ with a 5 mm membrane has a standard measurement range of 0–20.00 ppm, with a response time of 90% in less than 180 s. Sensitivity is 0.01 ppm with repeatability of ±0.05 ppm and an optional measurement range of 0–40.00 ppm is also available.

Because there is always direct contact between the sensor membrane and the liquid media being measured, there is a uniform oxygen transfer resulting in a true read-out of dissolved oxygen content. In addition, more accurate and consistent monitoring is achieved because the unit is also programmed to automatically clean itself, reducing maintenance procedures and increasing performance.

Cleaning of the sensor *in situ* is achieved with a blast of high-pressure air from an internal compressor. This removes biological growth and other contaminants from the surface of the sensor membrane. The cleaning frequency can be programmed from as little as once daily up to once every 2 h to cater for varying conditions. Each cleaning cycle lasts approximately 3 min, during which time the monitor output and alarm contacts are held at pre-cycle values to eliminate false dissolved oxygen readings being registered. This also prevents inadvertent triggering of an alarm relay which actuates on either low or high dissolved oxygen levels, or the failure of the control system to maintain dissolved oxygen concentrations at predetermined values.

The cleaning procedure is both efficient and effective with nothing to clog, break or wear out. It eliminates the maintenance and repair work associated with other less

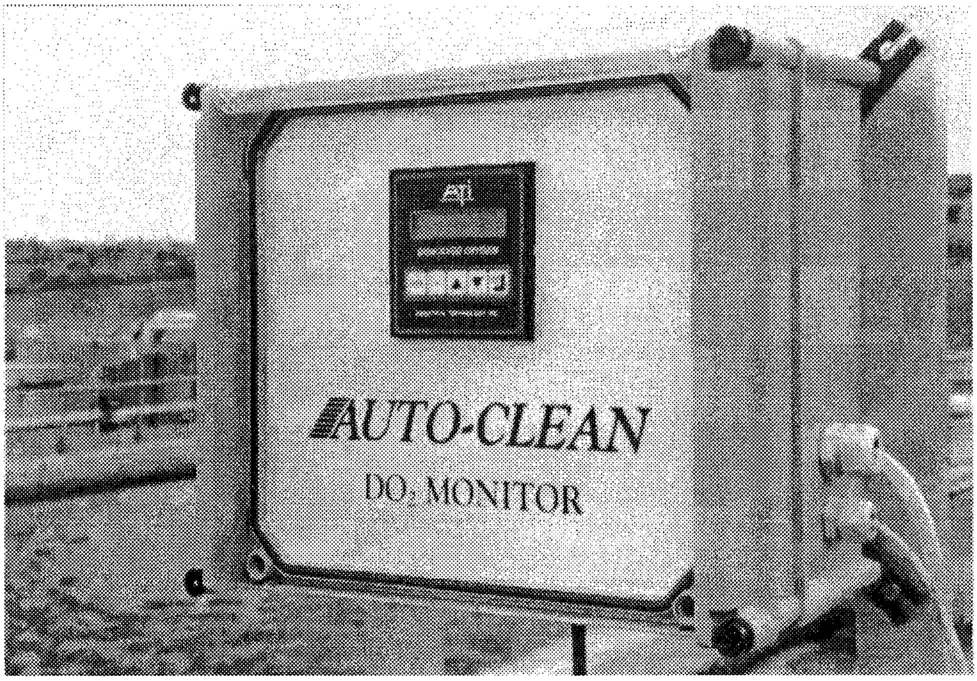

*The new Auto-Clean $DO_2$ dissolved oxygen monitor from ATi (UK) Ltd which features efficient and effective compressed air cleaning.*

effective cleaning systems which use grindstones or brushes. It also eliminates contaminant build-up which cannot be successfully removed by chlorine gas treatments as employed with some other manufacturers' products.

Installation of the equipment is both quick and simple. Special adaptors are supplied for connecting the sensor to standard 1″ conduit or pipe and then clamping it securely to a standard handrail system.

The NEMA 4X monitor and cleaner package can also be handrail-fitted using supports provided, or wall mounted if more convenient. Power requirements are 110Vac at 50/60 Hz, 300Va maximum with an optional 220Vac model also available. Operating temperatures are −20°C to +52°C, with the sensor rated for 0–50°C. Operating humidity is 0–99% non-condensing.

Further information on the Auto-Clean $DO_2$ is available on request to: ATi (UK) Ltd, First Floor, 237–239 Oldham Road, Springhead, Oldham OL4 4QL, UK. Telephone: 0161 624 0200; Fax: 0161 624 0400.

### New software package enables chemists to combine structural drawing with spectral interpretation

Chemists can now predict C-13 NMR shifts, MS fragmentation patterns and IR band correlations from a chemical structure using a single software package.

Sadtler Suite™ combines the latest versions of IR SearchMaster, IR Mentor and ChemWindow to allow retrieval, interpretation and presentation of results, working from either a drawn structure or spectral data.

By seamlessly integrating prediction, search and drawing software, Sadtler Suite enables chemists to annotate and publish spectral data, chromatograms and peak tables in reports alongside chemical structures.

An IR database, containing 2500 verified compounds, is included in the package to save time when finding matching spectra. IR Mentor Pro—with over 200 functional groups and 700 interpretation frequencies—provides a powerful tool for speeding up IR analysis.

Chromatograms, IR, NMR and MS spectra can be imported into ChemWindow Spectroscopy, to annotate with structures, peak tables and other custom features. ChemWindow also offers comprehensive drawing capabilities to create 3D presentations and 2D structures.

Sadtler Suite is the first software package to enable the seamless integration of all these processes with the ability to combine data from many different laboratory instruments.

For further information, contact: Peter Sandbach, HCC·De Facto. Tel: +44 (0)1256 842274; e-mail: P.Sandbach@defacto.co.uk

Deborah Kernan, Bio-Rad. Tel: +1 215 249 7366: e-mail: deborah_kernan@bio-rad.com; website: http://www.sadtlersuite.bio-rad.com

### An 'electronic nose' enables more consistent quality of packaging boards

A new instrumental analysis system, which evaluates the sensory properties of packaging boards, is intended to increase the accuracy and efficiency of the paperboard quality control at Finnish Enso Paperboards.

The system, called electronic nose, will be taken into operation in 1998 as a further quality control tool at the company's Imatra mill. It is a development of an

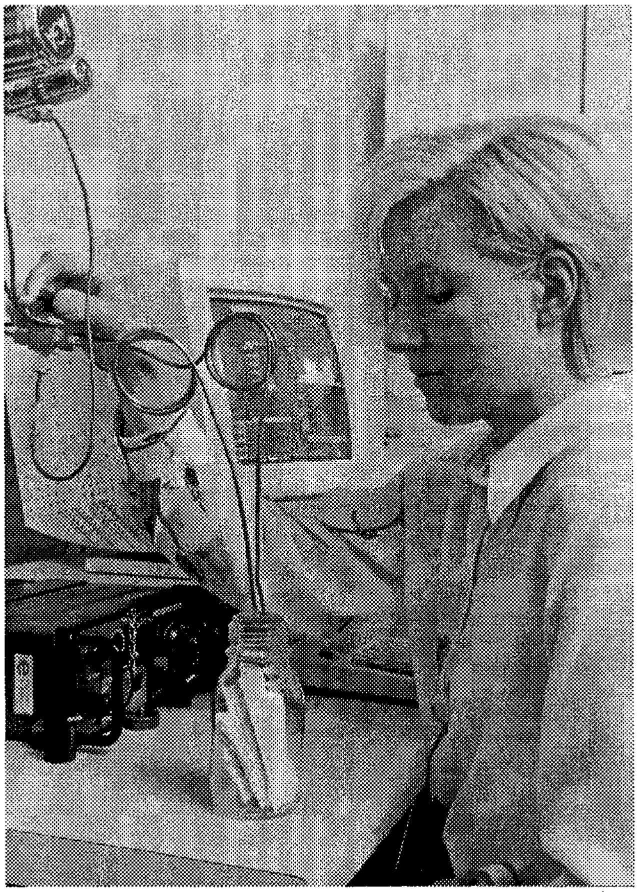

*A new instrumental analysis system, called the electronic nose, will be put into operation by Finnish Enso Paperboards in order to guarantee consistent quality of packaging paperboards.*

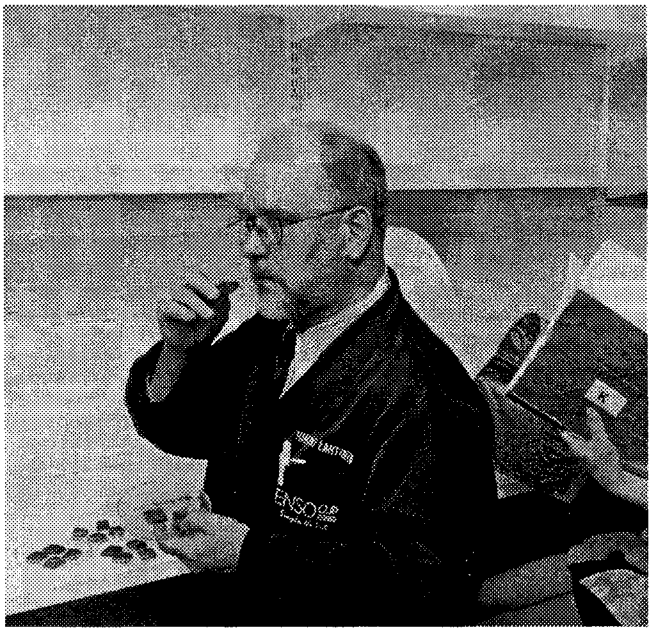

*The electronic nose will complement regular test methods, e.g. sensory test panels, says chemist Tarja Miettinen, responsible for the laboratory analyses.*

instrument, MDG-1, which was originally constructed in Finland for military use to detect chemical warfare agents.

To a great extent, the analysis system will be used for the company's Enso Performa paperboard, which is aimed at food, sweets and tobacco applications with high demands on odour and taste neutral packagings.

"The electronic nose mimics biological olfactory systems by processing signals from different kinds of sensors with sophisticated software. Unlike the human sense of smell, instrumental sensors are objective and resistant to fatigue. This makes the quality control system simpler and also more accurate and efficient", says Dr Henry Lindell at Enso Research Centre.

The new analysis system will be used as a complement to the present measurement methods, e.g. sensory evaluation by trained test panels, the Robinson test and gas chromatographic analyses. More advanced analyses, using GC/MS (gas chromatograph and mass spectrometer), are carried out at Enso Oyj's Research Centre in Imatra.

"The basic idea in the use of the electronic nose is to teach the system the typical odour of each board grade. After the teaching process, the electronic nose recognizes samples having different chemical characteristics from teaching material", explains Dr Henry Lindell.

The results of several months' research work seem promising. The correlation between sensory results by human test panels and the results of the electronic nose have gradually been improved by fine tuning of the electronic nose.

For further information, please contact: Enso Marketing Co., Enso House, New Mill Road, GB-ORPINGTON, Kent BR5 3QA, UK. Tel: +44 1689 836 911; Fax: +44 1689 829 732.

Enso (Middle East), P.O.B. 61265, Dubai, United Arab Emirates. Tel: +971 4 836 819; Fax: +971 4 836 826. (Bahrain, Cyprus, Iran, Iraq, Jordan, Kuwait, Lebanon, Oman, Qatar, Saudi Arabia, Syria, Yemen)

Enso-Eurocan Hong Kong Ltd., Rm. 1406 14th Floor, World Trade Centre, 280 Gloucester Road, Causeway Bay, Hong Kong, P. R. China. Tel: +852 2895 2063; Fax: +852 2576 1480.

Enso-Eurocan Hong Kong Ltd., Beijing Office, Rm. 808 Scitech Tower, 22 Jianguomenwai Avenue, Beijing 100004. Tel: +86 10 6515 7178; Fax: +86 10 6515 7179.

Enso-Eurocan South East Asia Pte. Ltd., Representative Office, Block 154-2-11, Kompleks Maluri, Jalan Jejaka, 55100 Kuala Lumpur. Tel: +60 3 981 8122; Fax: +60 3 987 3122.

Enso-Eurocan South East Asia Pte. Ltd., 360 Orchard Road, # 11-01 International Building, SG-Singapore 238869. Tel: +65 235 3485; Fax: +65 737 4278.

Enso-Eurocan Far East Co. Ltd., KM Nishiumeda Bldg., 20-1, Fukushima 7-chome, Fukushima-ku, J-Osaka 553. Tel: +81 6 455 6811; Fax: +81 6 455 8811.

Enso Italia S.r.l., Piazza S. Camillo de Lellis, 1, I-20124 Milan. Tel: +39 2 670 871; Fax: +39 2 669 7516.

Fins Verkoopkantoor/Enso (Holland) BV, Postbus 12386, 1100 AJ Amsterdam. Tel: 020-6505555; Fax: 020-6505622.

Enso International, 281 Tresser Blvd., 15th Floor, Stamford, CT 06901. Tel: +1 203 978 1755; Fax: +1 203 978 1237.

Enso International, 2550 Leavenworth St., Suite 2, San Francisco, CA 94133. Tel: +1 415 567 1295; Fax: +1 415 567 1281.

Enso Interamericas, 8750 Doral Boulevard, Suite 270, Miami, FL 33178. Tel: +1 305 716 9799; Fax: +1 305 716 5441.

T.S.P., 2 Main Street, Chatham, New Jersey 07928. Tel: +1 201 635 3530; Fax: +1 201 635 1680.

Paper Agencies (Aust.) Pty. Ltd., 554 Burwood Road, Hawthorn, Vic. 3122. Tel: +61 3 9819 4911; Fax: +61 3 9819 4250.

Paper Agencies (Aust.) Pty. Ltd., Suite 5, 102 Alfred Street, Milsons Point, NSW 2061. Tel: +61 2 9957 3177 Fax: +61 2 9957 1505.

Enso France S.A., 15/25 Boulevard Amiral Bruix, F-75782 Paris Cedex 16. Tel: 01 53 64 79 00; Fax: 01 53 64 79 90.

S.A. Comptoir Finlandais N.V., Avenue des Gaulois 7, B-1040 Bruxelles. Tel: 02 743.13.60 Fax: 02 736.01.78.

Enso (Schweiz) AG, Bahnhofstrasse 13, CH-8808 Pfäffikon SZ. Tel: 055 415 3060; Fax: 055 415 3069. (Egalement pour le Liechtenstein)

Enso (Deutschland) GmbH, Große Bleichen 30, D-20354 Hamburg. Tel: 040 350 990; Fax: 040 3509 9146.

Enso Española S.A., Apartado 76 – 08760 Martorell, Barcelona. Tel: 93 631 1000; Fax: 93 631 1021.

Enso Sverige AB, Joakim Stockhaus, försäljningsdirektör, Box 2017, SE-750 02 Uppsala, Sweden. Tel: 018 65 30 00; Fax: 018 65 30 10. E-mail: oakim.stockhaus@up.mkt.enso.com

### News

### Solutia reviewing options for phosphorus derivatives business

*St. Louis, 1 July 1998:* Solutia (NYSE:SOI) said today that it is reviewing options for its phosphorus derivatives business, including sale, alliance and joint venture, and has retained the investment banking firm Goldman Sachs to assist in the evaluation.

"The phosphorus derivatives business has been profitable for Solutia," said Michael E. Miller, Solutia vice chairman, "but it's clear that global consolidation in some form in this industry is likely to happen. To fulfill management's commitment to the Board of Directors and to our stockholders, it's imperative that we explore value-enhancing opportunities for our businesses".

### News from the September issue of Performance chemicals international

*Surfactants*: It is a time of change in the surfactants sector. Increasingly, demanding customer requirements in both the industrial and personal care areas, as well as the growing importance of environmental considerations, are encouraging change right across the sector. Mirroring the trend in the rest of the industry, M&A activity has been intense over the past year or so as we move towards fewer but larger players. The sector is consolidating in an attempt to improve its profitability, a phenomenon seen throughout the speciality sector which is making life harder for the smaller player.

*Food additives*: The market for food additives is characterized by increasing sophistication with demand mainly propelled by the rapidly growing processed foods sector. As lifestyles change and food processing technology evolves, there is increasing demand for a variety of food additives, ranging from sweeteners to thickeners. 'Consumer preference in foods and flavours is continually changing due to fashion, lifestyle changes and the focus on healthy eating,' says Manoj Gujral, business director and general manager at Oxford Chemicals, a company that specializes in the manufacture and supply of speciality aroma chemicals to the global market for flavours and fragrances.

*Ireland*: Ireland is becoming a key location for the European pharmaceutical and chemical industry. More than 120 overseas companies employ 15 000 people in this sector in Ireland. In 1997, pharmaceutical products worth more than $7bn were exported. This represents 15% of total exports and makes Ireland one of the world's largest exporters of pharmaceuticals and fine chemicals—sectors that dominate the Irish chemical industry. The heavy and petrochemical industry is not well represented due to a lack of raw materials and the capital-intensive nature of this end of the industry.

*Catalysts*: Catalysts remain one of the more innovative areas of chemistry with new technologies being developed that could have dramatic impacts on he economics of chemical processes worldwide. In the USA, a researcher has suggested a method of using combinatorial chemistry to discover new solid-state catalysts. In a paper published in the latest edition of *Nature* magazine, Selim Sankan of the chemical engineering department at the University of

California, Los Angeles, suggests a 'mass screening' technique that could speed up the current trial and error process. The pharamaceutical industry already uses combinatorial synthesis methods, in combination with mass-screening techniques, to speed up the discovery of new drugs.

*Technical alert*: A US consortium has announced what it describes as a 'a major milestone' in the development of biotechnological routes to chemicals from renewable resources. The consortium members are Genencor International, Eastman Chemical, Electrosynthesis Company, MicroGenomics and Argonne National Laboratory, a Department of Energy (DOE) lab. The first target chemical for the process is ascorbic acid, which has a world market worth $600m. The consortium says the process could cut the capital cost by half compared to the conventional Reichstein process.

For more information, call the Editor Alan Tyler at 44-181-652 8126, email alantyler@rbi.co.uk.

- *Performance Chemicals International* is published by the Reed Chemical Group, part of Reed Business Information (RBI). The Reed Chemical Group also includes *Asia-Pacific Chemicals, Asian Chemical News, European Chemical News, Chemical Insight* and 24 h online service Chemical News and Intelligence (CNI).
- RBI publishes over 60 titles including: *New Scientist, Flight International, Estates Gazette, Contract Journal, Commercial Motor, Doctor, Bankers Almanac, Kelly's Business Directory, Kompass British Exports* and many more. For a full listing see RBI's web site http://www.reedbusiness.com

### Top five chemical companies generated $134.6 bn in sales in 1997, new analysis shows

The five largest chemical companies in the world generated sales of $134.6 billion in 1997 and profits before tax totalling $15.5 billion, according to *Chemical Insight*'s analysis of annual financial performance in the chemical industry. Combined net profits for the top five were $7.7 billion.

The 1997 analysis puts four US companies at the head of the performance league table—Union Carbide, Georgia Gulf, Rohm & Haas, and Nalco. These are followed by one of Europe's largest petrochemical producers, Borealis, and Britain's pharmaceuticals, agrochemicals and speciality chemicals producer, Zeneca. The chemical industry is heading for hard times, but the analysis for 1997 shows just how much stronger some of the European producers have become and their good standing—in 1997 at least—among their peers.

This critical comparison looks at the performance of companies across the globe. It assesses performance according to 32 financial measures and ratios, and looks at the industry in ways which compare year-on-year performance, profitability, productivity and financial health. The analysis is all about comparisons across national boundaries, regions and accounting conventions. It shows which companies performed well in 1997 and points to future strengths.

The analysis issue contains detailed financial information on the top 30 companies in the chemical industry. It ranks companies by sales and gives net profits and net margins, and research and development and capital spending figures. Productivity is an important element of the analysis, and the productivity data published rank companies by sales per employee and give figures for value added, employee numbers and employee costs.

The industry is facing difficult times now, and Union Carbide is not alone is issuing a third quarter 1998 profits warning. However, average industry sales rose by 7% last year. Cash flows were 9% higher and pre-tax profits up by 12%.

Chemical Insight *is published by Reed Chemical Publications in the UK, Reed Business Information (RBI), a division of Reed Elsevier. Other chemical publications published by RBI include* European Chemical News, Asia Pacific Chemicals, Performance Chemicals International, *and* Chemical News & Intelligence, *a worldwide, Internet-based 24 h news service. Reed Business Information also publishes over 60 titles, including:* Flight International, Estates Gazette, Contract Journal, Commercial Motor, Doctor, Bankers Almanac, Kelly's Business Directory, Kompass British Exports, *and many more. For a full listing see RBI's web site—http://www.reedbusiness.com. For further details about* Chemical Insight, *contact the Editor, Nigel Davis, e-mail: nigel.davis@rbi.co.uk, Tel: +44 (0)181 652 3397, Fax: +44 (0)181 652 8952*

### Patent application fees abolished

Small firms and private individuals benefitted most when the United Kingdom Patent Office abolished the application fee for patents from 1 October 1998. The Office is the first in the industrialized world not to charge a patent application fee.

Abolition of the filing fee forms part of a 20% cut in Patent Office fees that came into effect on 1 October. Other reductions include a cut in the trademark application fee from £225 to £200, a cut in trademark registration renewal from £250 to £200, and cuts in patent renewal fees by an average of 18%.

The cuts assist entry into the systems of patents, trademarks and registered designs, and encourage their use by small firms and private individuals. The greatest savings to be made are in patent renewals in the earlier years, when companies are frequently still in the phase of product development and have yet to make a return on their investment. Savings to industry will equal £12 million, or 20% of the Patent Office's fee income.

Welcoming the fee reductions, Patent Office chief executive Paul Hartnack said: 'The reductions in Patent Office fees make a significant contribution to the competitiveness of British industry, particularly among the small firms which are the source of innovation and creativity in Britain'.

'The fee reductions have been made possible by the continuous and successful efforts of management and staff to raise the quality of service and reduce unit costs since the Patent Office relocated to Newport, South Wales, in 1991'.

Details of the fee reductions are available on the Patent Office Web site at www.patent.gov.uk/snews/notices/red-fee.html. Copies of the statutory instruments are available from the Stationery Office Bookshops and from the Patent Office. They are *The Trade Marks (Fees) Rules 1998* (SI 1998 No 1776), £1.10; *The Registered Designs (Fees) Rules 1998* (SI 1998 No 1777), £1.10 and *The Patents (Fees) Rules 1998* (SI 1998 No 1778), £1.95).

*For information, contact Brian Caswell, The Patent Office, +44 (0)1633 814729 or Michael Binns, Prowse & Co, +44 (0)1372 363386.*

### 21st century requirements to monitor our environment prompts action by The Royal Society of Chemistry

Modern instrumentation has allowed us to push back the frontiers of detection such that we are able to determine incredibly small amounts of natural and anthropogenic pollutants and contaminants in our environment, whether they are in our homes, workplaces, cities, the countryside or the oceans. The fact that we can detect these pollutants in minuscule amounts does not necessarily mean that the levels present in the environment are harmful to our health or well being, but it does drive world-wide legislation on these substances. Therefore, there is a requirement to monitor, ascertain the sources, prevent the release, develop better detection methods and make properly assessed scientific judgements on the toxicity, exposure and risk assessment of the pollutants to which we are exposed in our daily lives.

The Royal Society of Chemistry has recognized the importance of these 21st century requirements, and that it is essential to promote and disseminate the knowledge of newly developed technologies for monitoring our various environments. Therefore, it is launching the *Journal of Environmental Monitoring (JEM)* which is dedicated to assessing exposure and health risks through the latest developments in measurement science. The journal, with the first issue due to be published in February 1999 and then bi-monthly thereafter, is unique in that it aims to publish all the relevant information on this subject area in one source.

This journal is intended for environmental and health professionals in industry, and officials from governmental and regulatory agencies as well as research scientists interested in the environment.

"I think the journal will be of interest to all analytical scientists involved with environmental monitoring issues. Currently at NIOSH, environmental monitoring is one of the key components of the National Occupational Research Agenda (NORA)". Dr E. R. Kennedy, NIOSH, Cincinnati, USA.

"Environmental contaminants are becoming the No. 1 public concern". Mr J. V. Dutton, Consultant, UK.

"The launching of a journal dedicated to environmental monitoring with some emphasis on legislative issues is an excellent idea". Dr. Philippe Quevauviller, European Commission, DGXII SM&T Programme, Belgium.

The Royal Society of Chemistry (RSC) is a learned society with a worldwide membership of 46 000. it has as its main objectives the advancement of the science of chemistry and its applications, and the maintenance of high standards of competence and integrity among practising chemists. The RSC markets a comprehensive range of high quality information products and services.

For further details contact: Harpal Minhas, The Royal Society of Chemistry, Thomas Graham House, Science Park, Milton Road, Cambridge CN4 4WF, UK. Tel: +44 (0)1223 432292; Fax: +44 (0)1223 420247; e-mail: jem@rsc.org; URL: www.rsc.org/jem

### Meetings reports

### Micromass host international seminar on the enabling role of MS

Micromass, the mass spectrometry people, hosted *MS in Proteomics & Drug Discovery: An International Seminar on the Enabling Role of MS.* The seminar, which was open to the public and incorporated Micromass' 1998 organic MS users' meeting, the first event of its kind to be organized by the company since its re-formation in 1996.

The seminar saw a move from central Manchester, the traditional home of the Micromass user meeting, to Shrigley Hall in Cheshire. From 18th to 20th October 1998, this impressive 19th century country mansion housed an international panel of invited speakers and Micromass users, presenting an insight into emerging MS-based strategies in proteomics and contemporary drug discovery/development. Speakers included Roland Annan (SmithKline Beecham, PA, USA), Walter Blackstock (Glaxo Wellcome, UK), Daryl Pappin (Imperial Cancer Research Foundation, UK) and Jeffrey Kiplinger (Pfizer, Groton, CT, USA).

Originally planned as a 'stand-alone' meeting to be hosted, earlier in 1998, by Glaxo-Wellcome, the proteomics sessions were, through unexpectedly popular demand, rescheduled at this larger venue. Protein identification using MS and bio-informatics is becoming routine in the quest for new targets, with known proteins being rapidly identified via comparison of MS data with genome, EST or protein databases. Unknown proteins require the use of partial sequencing by MS-MS for the construction of oligonucleotide probes or primers. Additionally, *de novo* sequencing of peptide fragments is being automated. Initially focusing on current achievements in protein characterization using Q-Tof™ MS-MS systems, the intention of this seminar was to present an assessment of what was currently achievable, the limitations and what problems remained to be solved.

Moving onto the pharmaceutical industry, the seminar investigated one of the most important changes taking place within it, i.e. the rapid expansion of discovery operations. MS contributes to this process in several ways, including the provision of structural support for synthetic chemistry, autopurification prior to screening and LC-MS in lead optimization. LC-MS(MS) is now being used to evaluate drug candidate metabolism and pharmacokinetics at both the early and late development stages. Discovery teams are increasingly being requested to focus on the survival of candidates to and beyond first testing in human subjects, touching off a strong push to move testing traditionally done in a development setting into the high throughput early discovery environment.

LC-TOF MS mass analysers (LCT/Q-Tof) were highlighted in view of their significant potential to leverage throughput and data quality in these challenging applications.

*For further information, contact Dawn Eaton, Micromass UK Limited, 3 Tudor Road, Altrincham, Cheshire WA14 5RZ, UK. Tel: +44 (0)161 282 9666. Fax: +44 (0)161 282 4400. E-mail: dawn.eaton@micromass.co.uk*

### ISLAR '98

Cooler temperatures and changing leaves not only signal the annual arrival of autumn, but also the return of the International Symposium on Laboratory Automation and Robotics (ISLAR). On 18–21 October 1998, the world's leading scientists and managers convened in Boston, MA, for an innovative experience at the 16th annual ISLAR symposium.

With over 130 podium and poster presentations, ISLAR '98 is the world's largest forum to learn about the latest developments in automation and robotics. Its unique format features both management and technical presentations, making it the most important industry-related conference of the year. World-renowned scientists and managers in the biotechnology, pharmaceutical, chemical and consumer products industries shared their insight on the importance of using the latest technology and strategies to increase productivity and reduce time to market.

The three-day program included a series of discussion groups, workshops, short courses and presentations developed for managers to exchange ideas and experiences. A new feature at ISLAR '98 was an Ultra High Throughput Screening session where scientists and managers from around the world discussed industry challenges, the new era of HTS, and how automation was being used as a critical component in the advancement of high-throughput screening.

Attendees and presenters at ISLAR '97 participated in a survey which resulted in several new sessions debuting at ISLAR '98, including the following.

- Assay miniaturization technologies
- The role of bioinformatics in pharmaceutical research
- The impact of automation on secondary screening
- Managing laboratory automation in pharmaceutical analysis
- Methods transfer and validation
- Inhalation applications

To help members of the media maximize their experiences at the symposium, ISLAR '98 featured a series of editorial roundtables focusing on drug discovery, analytical research and development, and quality control/ quality assurance. The roundtable panels featured keynote speakers and symposium presenters to help facilitate discussions.

*For more detailed information and abstracts, access the ISLAR web site at http://www.islar.com. The next issue of* Journal of Automated Methods & Management in Chemistry *will feature abstracts from ISLAR '98*

